# Study on prevention of hypercapnia by Nasal High Flow in patients with endoscopic submucosal dissection during intravenous anesthesia

**DOI:** 10.1097/MD.0000000000020038

**Published:** 2020-05-08

**Authors:** Takao Ayuse, Naoyuki Yamguchi, Keiichi Hashiguchi, Takuro Sanuki, Gaku Mishima, Shinji Kurata, Naoki Hosogaya, Sawako Nakashima, Max Pinkham, Stanislav Tatkov, Kazuhiko Nakao

**Affiliations:** a Division of Clinical Physiology, Department of Translational Medical Sciences; b Department of Gastroenterology and Hepatology, Nagasaki University Graduate School of Biomedical Sciences; c Department of Dental Anesthesiology, Nagasaki University Hospital; d Nagasaki University Hospital, Clinical Research Center, Nagasaki, Japan; e Fisher & Paykel Healthcare Ltd, Auckland, New Zealand.

**Keywords:** hypercapnia, intravenous sedation, nasal High Flow

## Abstract

**Background:**

For relatively invasive upper gastrointestinal endoscopy procedures, such as an endoscopic submucosal dissection (ESD), intravenous anesthesia is routinely used to reduce patient anxiety. However, with the use of intravenous sedation, even at mild to moderate depth of anesthesia, there is always a risk of upper airway obstruction due to a relaxation of the upper airway muscles.

With the advent of Nasal High Flow (NHF) devices that allow humidified high flow air through the nasal cavity, can be used as a respiratory management method in the context of anesthesia. AIRVO is commonly used for patients with obstructive sleep apnea and other respiratory disorders. This device uses a mild positive pressure load (several cmH_2_O) that improves carbon dioxide (CO_2_) washout and reduces rebreathing to improve respiratory function and therefore is widely used to prevent hypoxemia and hypercapnia.

This study aims to maintain upper airway patency by applying NHF with air (AIRVO) as a respiratory management method during intravenous anesthesia for patients undergoing an ESD. In addition, this study investigates whether the use of an NHF device in this context can prevent intraoperative hypercapnia and hypoxemia.

**Methods/design:**

This study design employed 2 groups of subjects. Both received intravenous anesthesia while undergoing an ESD, and 1 group also used a concurrent nasal cannula NHF device. Here we examine if the use of an NHF device during intravenous anesthesia can prevent hypoxemia and hypercapnia, which could translate to improved anesthesia management.

Efficacy endpoints were assessed using a transcutaneous CO_2_ monitor. This device measured the changes in CO_2_ concentration during treatment. Transcutaneous CO_2_ (PtcCO_2)_ concentrations of 60 mmHg or more (PaCO_2_ > 55 mmHg) were considered marked hypercapnia. PtcCO_2_ concentrations of 50 to 60 mmHg or more (equivalent to PaCO_2_ > 45 mmHg) were considered moderate hypercapnia.

Furthermore, the incidence of hypoxemia with a transcutaneous oxygen saturation value of 90% or less, and whether the use of NHF was effective in preventing this adverse clinical event were evaluated.

**Discussion:**

The purpose of this study was to obtain evidence for the utility of NHF as a potential therapeutic device for patients undergoing an ESD under anesthesia, assessed by determining if the incidence rates of hypercapnia and hypoxemia decreased in the NHF device group, compared to the control group that did not use of this device.

**Trial registration:**

The study was registered the jRCTs 072190022.

URL https://jrct.niph.go.jp/en-latest-detail/jRCTs072190022

## 1 Introduction

For relatively invasive upper gastrointestinal endoscopy procedures, such as an endoscopic submucosal dissection (ESD), intravenous anesthesia is routinely used to reduce patient anxiety. However, with the use of intravenous anesthesia, even at mild to moderate depth of anesthesia, there is always a risk of upper airway obstruction due to a relaxation of the upper airway muscles. “Guidelines on sedation in endoscopic practice” report on respiratory depression during an ESD, and how it relates to sedation.^[1,2]^ It has been reported that the frequently used intravenous anesthesia method of 4 to 5 mg of midazolam and 70 to 100 mg of pethidine hydrochloride during an ESD is associated with the occurrence of respiratory depression at rates as high as 85%.^[3]^

Interestingly, it has been reported that respiratory complications that occur during intravenous anesthesia have a higher risk of hypercapnia than hypoxemia.^[1,4–7]^

Moreover, when low-flow oxygen is administered through a nasal cannula, the apparent percutaneous oxygen saturation value is maintained at a normal concentration, however hypoventilation is sustained, which may result in impaired exhalation. The conditions of a gradual CO_2_ accumulation, and also CO_2_ concentrations maintained at 60 mmHg or higher, are risk factors for secondary circulatory abnormalities such as an abnormal increase in blood pressure, tachycardia, and arrhythmia. In addition, patients treated for ESD often have chronic obstructive pulmonary disease (COPD), which can lead to significant carbon dioxide accumulation depending on the severity of COPD.

Furthermore, methods of insufflating CO_2_ into the intestinal tract concurrently with ESD have been widely used in place of conventional air insufflation in the recent years; it has been pointed out that this may reduce the pain experienced by the patient. However, it has also been reported that the intestinal tract absorbs CO_2_ transiently, and it is necessary to pay close attention to signs of the occurrence of hypercapnia. In a study that involved the measurement of transcutaneous carbon dioxide (PtcCO_2_) during colon ESD,^[2]^ the peak mean value of PtcCO_2_ was 55.6 mmHg for the CO_2_ insufflation group and arrhythmia could occur. In this study, eight of the 37 cases (22%) had a PtcCO_2_ value of 60 mmHg or higher. For gastric and esophageal ESD, PtcCO_2_ was higher than 60 mmHg for more than 5 minutes in 64 cases, of which 10 cases (16%) were observed in the CO_2_ insufflation group and 5 cases were observed in the air insufflation group. Data on 104 ESD patients under intravenous anesthesia managed by an anesthesiologist^[8]^ revealed an increase from an average of 44 mmHg to 60 mmHg for the CO_2_ insufflation group. It was reported that CO_2_ accumulation could not be prevented even if a physician managed it.^[9]^

Hence, preventing transient or continuous CO_2_ accumulation during examinations and procedures performed under intravenous anesthesia is critical for safe respiratory management.

Therefore, it is extremely important to monitor and prevent transient or continuous CO_2_ accumulation under intravenous anesthesia to maintain safe respiratory management.

A candidate to further improve respiratory management under intravenous anesthesia, is the use of a device called Nasal High Flow (NHF), which provides humidified air to flow to the nasal cavity at a high flow rate. NHF is typically used in patients with obstructive sleep apnea syndrome and respiratory disorders. This device has begun to be widely applied to the prevention of hypoxemia. It has a mild positive pressure load (several cmH_2_O) that improves respiratory function in part by improving CO_2_ wash out and reducing CO_2_ rebreathing.^[10–12]^ In addition, most recently, improved respiratory management using NHF is being studied during the propofol sedation.^[13]^ The hypothesis is that NHF use could prevent and resolve the onset of hypercapnia associated with transient upper airway obstruction during procedural sedation.

The purpose of this study was to maintain upper airway patency by applying NHF as a respiratory management method during intravenous sedation to patients undergoing ESD performed under intravenous sedation. In addition, this study investigates whether improving respiratory management could prevent intraoperative hypercapnia and hypoxemia.

## 2 Methods/design

### 2.1 Study design

The present study was designed in accordance with the Standard Protocol Items: Recommendations for Interventional Trials and Consolidated Standard of Reporting Trials 2010 guidelines.^[14,15]^

This was an open-label, investigator-initiated, single center study on the efficacy of NHF use in patients undergoing ESD performed under intravenous anesthesia. Treatment schedule and outcome measures is shown Table 1.

**Table 1:**
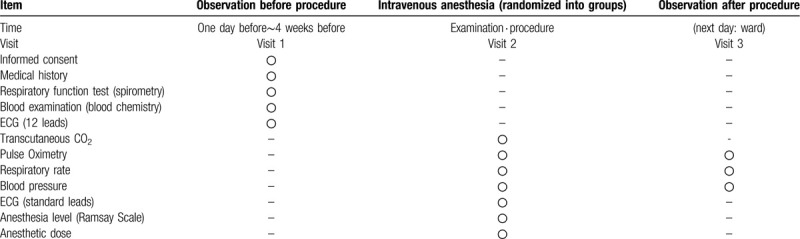
ESD time schedule.

The Clinical Research Review Board in Nagasaki University approved the study and study protocol. The study was conducted at Nagasaki University Hospital in Japan. The study is registered on the jRCTs. The study was conducted in accordance with the principles of the Declaration of Helsinki and the established best clinical practices of Japan.

### 2.2 Participant recruitment

Participants were recruited from the Nagasaki University Hospital, where the subsequent study also took place. The treating clinical research coordinator (CRC) provided an explanation of the study to all participants, and these participants signed an informed consent form. This was a randomized control study, comprised of 2 groups of participants. While both groups received intravenous anesthesia during ESD, one group concomitantly used an NHF device. Allocation method: Research Electronic Data Capture (REDCap) was used to randomly allocate participants to “NHF-using” and “non-NHF-using” groups at a ratio of 1:1 (stratified block method). Allocation factor: Allocation was based on the presence or absence of COPD. If a participant had a history of smoking and the respiratory function test (spirogram) recorded a 1-second rate of less than 70%, it was determined that COPD was present and allocation was performed.

### 2.3 Inclusion criteria

The following were inclusion criteria: Adult patients between the ages of 20 and 85, that gave informed consent after a thorough explanation of all details of this clinical trial.

### 2.4 Exclusion criteria

The exclusion criteria were as follows:

(1) continuous administration of oxygen by nasal cannula (home oxygen therapy),
(2) inability to breathe through the nose,
(3) use of antithrombotic drugs that could not be reduced or discontinued on the day before the endoscope,
(4) a history of pneumothorax,
(5) judged inappropriate as study subjects.

### 2.5 Study protocol

All participants underwent an ESD under intravenous anesthesia, in a supine position on an examination table, in an endoscope room. Patients assigned to the NHF device group were cannulated with NHF in both nasal passages and provided with humidified air at a flow rate of 40 to 60 L/min.

A blood pressure monitor, electrocardiogram, and percutaneous oxygen saturation probe attached to the finger were used at the time of examination and treatment. Moreover, a transcutaneous CO_2_ (PtcCO_2_) probe was attached to the anterior chest or medial arm, and vital signs were continuously monitored. In order to continuously and non-invasively evaluate the circulating CO_2_ concentration, a PtcCO_2_ concentration that was not affected even when inhaling high-flow air with a nasal cannula was used. PtcCO_2_ was measured by a continuously attached electrode of the transcutaneous CO_2_ monitor (Radiometer, Inc, Japan) to the anterior chest and measuring the PtcCO_2_ concentration together with the transcutaneous oxygen saturation and respiratory rate. Values were obtained from the anesthesia record of the electronic medical record (Nihon Kohden, Inc, Japan). In the group receiving NHF, the values of vital signs 10 minutes after inhalation from the nasal cannula were recorded.

In the detection of the respiratory waveform, the respiratory phase, and the respiratory rate were evaluated based on the change in impedance between the electrodes using the three-electrode standard lead of the electrocardiogram.

To evaluate efficacy of NHF in the prevention of hypercapnia, the transcutaneous CO_2_ monitor output was used. PtcCO_2_ concentrations of 60 mmHg or more (PaCO_2_ > 55 mmHg) were considered marked hypercapnia. PtcCO_2_ concentrations of 50 to 60 mmHg (equivalent to PaCO_2_ > 45 mmHg) were considered moderate hypercapnia. Furthermore, the incidence of hypoxemia with a transcutaneous oxygen saturation value of 90% or less was evaluated. Results of these parameters determined whether the use of the NHF was effective in preventing the development of hypercapnia and hypoxemia.

Another parameter of the study that was assessed was the respiratory rate, evaluated by the change in impedance of an electrocardiographic (ECG) electrode. The depth of anesthesia was evaluated using the Ramsay scale, along with the total dose of the anesthetic and its dose per time.

In the NHF device group, patients used AIRVO2 (manufactured by Fisher & Paykel Healthcare, Auckland, New Zealand) to inhale humidified air at a flow rate of 40 to 60 L/min from a nasal cannula.

### 2.6 Adverse events

In this study, the NHF device was secured to the patient via a nasal cannula, and this might have felt uncomfortable. Moreover, an electrode for transcutaneous carbon dioxide concentration measurements was attached to the anterior chest for both the NFH group and non-NHF group. For participants with sensitive skin, this might have caused temporary redness. When these adverse events occurred, care was taken to prevent undue harm to the research subjects.

### 2.7 Outcome

The primary endpoint was:

(1) The occurrence rate of severe hypercapnia with a maximum PtcCO_2_ concentration of 60 mmHg or more (equivalent to PaCO_2_ > 55 mmHg) during intravenous sedation,
(2) Area under the curve of percutaneous CO2 concentration per unit time during intravenous anesthesia (AUC),
(3) Duration of moderate hypercapnia showing maximum PtcCO_2_ concentration of 50 mmHg or more (equivalent to PaCO_2_ > 45 mmHg) during intravenous sedation,
(4) Occurrence rate of hypoxemia with percutaneous oxygen saturation value of 90% or less during intravenous sedation.

### 2.8 Efficacy

We evaluated the efficacy of the investigational device on a number of parameters, including: changes in CO_2_ concentration during treatment, the rate of occurrence of marked hypercapnia in which the maximum value of PtcCO_2_ concentration is 60 mmHg or more (equivalent to PaCO_2_ > 55 mmHg), and the duration of moderate hypercapnia as defined by a PtcCO_2_ concentration of 50 to 60 mmHg (equivalent to PaCO_2_ > 45 mmHg). Additionally, the incidence of hypoxemia was evaluated as defined by a transcutaneous oxygen saturation value of 90% or lower.

### 2.9 Safety

The safety evaluation indices of this clinical trial are as follows: adverse events are any undesired or unintended signs (including abnormal laboratory values, abnormal vital signs), symptoms, or illnesses that occur between the start of medical device (NHF) use and the end of the last observational study. This does not matter whether the study has a causal relationship. Symptoms and diseases occurring before the use of medical devices are treated as complications and not adverse events. However, if the complications worsen after the date of starting medical device use, they will be treated as adverse events, and the day on which the deterioration is confirmed will be the date of occurrence of the adverse events.

### 2.10 Data collection and management

The assignment table and input table used in this study were created with Research Electronic Data Capture (REDCap). The study was conducted after allocating the registered patients, and the data of all items in the medical record collected in the study were assigned to the researcher assigned the ID entered by physician, co-doctor and co-worker. The Principal Investigator or Co-Researcher approved the input observation / inspection / evaluation data of each research subject immediately after confirming the content.

For the data entered in the case report, the Principal Investigator and the Clinical Research Center Data Management staff perform a visual check and a logical check. Consequent to each check, if there are any problems or doubts in the data, the principal investigator, or the research coordinator is contacted. The case is fixed by performing data lock on the case when the issue has been resolved, and any modifications have been completed. If there is an error that needs to be corrected after the case is locked, the data management staff is responsible for overseeing this process.

In this study, monitoring will be carried out in accordance with the research plan and monitoring procedures to ensure that the research is being conducted properly.

### 2.11 Statistical analysis

Since this study is exploratory, we estimated an incidence rate for each study parameter and then used this information to calculate the necessary sample size for the verification study to achieve statistical significance.

Specifically, the rate of occurrence of hypercapnia in the NHF device group and the control group were calculated, and the difference (ratio) between the 2 groups and the 95% confidence interval on both sides were calculated. For other items, the average value and standard deviation for each group were manually calculated.

## 3 Discussion

The goal of using an NHF device, during an ESD under intravenous sedation, was to prevent not only acute hypercapnia, but also hypoxemia. The clinical conditions resulting from a transient upper airway obstruction that can occur during intravenous anesthesia could be prevented or ameliorated by the use of an NHF device. It has been reported that the frequently used intravenous sedation during an ESD is associated with the higher occurrence rate of respiratory depression.^[3]^ Furthermore, it has been reported that respiratory complications that occur during intravenous anesthesia have a higher risk of hypercapnia than hypoxemia.^[1,4–7]^ Therefore, the prevention of hypercapnia could be important factor for achieving safe respiratory management during procedural sedation for ESD.

This is an exploratory study aimed at collecting information for conducting a verification study. Therefore, the number of cases is set based on the feasibility in our hospital.

In Nagasaki University Hospital, about 200 patients undergo esophageal / gastric ESD under intravenous anesthesia for 1 year. Approximately 100 cases with a data collection period of 6 months, of which approximately 70% can obtain consent, will be about 70 cases. The 72 ESD cases are assigned to 2 groups, a device use group (36 cases) and a non-use group (36 cases), and stratified by the assignment factor (presence or absence of COPD) (18 cases each). Similarly, in the case of colorectal ESD, 32 cases are assigned to 2 groups, a device use group (16 cases) and a non-use group (16 cases), and stratified (8 cases each) by an allocation factor (presence or absence of COPD). Furthermore, the incidence of hypercapnia in the target population is estimated to be around 10% to 20%. Therefore, the number of events in 1 group was 16 and the possibility of generating an event was increased. Therefore, we will enroll total 104 cases in this study (72 cases of esophageal / stomach ESD and 32 cases of colon ESD).

The primary endpoint of this study was:

(1) The occurrence rate of severe hypercapnia with a maximum PtcCO_2_ concentration of 60 mmHg or more (equivalent to PaCO_2_ > 55 mmHg) during intravenous anesthesia,
(2) Area under the curve of percutaneous CO2 concentration per unit time during intravenous anesthesia (AUC),
(3) Duration of moderate hypercapnia showing maximum PtcCO_2_ concentration of 50 mmHg or more (equivalent to PaCO_2_ > 45 mmHg) during intravenous anesthesia,
(4) Occurrence rate of hypoxemia with percutaneous oxygen saturation value of 90% or less during intravenous anesthesia.

In addition, the evaluation of depth of anesthesia using Ramsay scale and total anesthetic dose during sedation will be also be assessed as a secondary endpoint.

Schumann et al reported that the availability to use NHF during sedation in an endoscopy suite reduced the requirement for anesthesia to perform complex endoscopic procedures.^[16]^ NHF reduces the re-breathing of expired CO_2_ from the anatomical dead space, which allows for maintained gas exchange at a lower minute ventilation.^[17]^ Therefore, patients can achieve the same alveolar ventilation with a reduced workload for the respiratory muscles.^[18]^

The most recent our study indicated that during sedation with propofol, NHF without supplemental oxygen attenuates CO_2_ retention and reduces the respiratory rate.^[13]^ The findings suggest that NHF can improve ventilation during procedural sedation for relatively invasive upper gastrointestinal endoscopy procedures, such as an ESD, which may reduce the risk of complications related to hypoventilation.

## Acknowledgments

The authors would like to thank our colleagues and staff at the Dental Anesthesiology, Gastroenterology and Hepatology Department of Nagasaki University Hospital for their support.

## Author contributions

TA, TS, GM, SK, NH, and SN are responsible for conceiving and designing the trial, planning data analysis, drafting the manuscript, and approving the final manuscript. MP, and ST are responsible for preparing and completing set up of AIRVO device including all equipment. NY, and KH, will participate in data collection and are in charge of recruitment and treatment of patients. All authors will have access to the interim results as well as the capacity to discuss, revise, and approve the final manuscript.
